# Effect of Intermittent Compared With Continuous Energy Restricted Diet on Glycemic Control in Patients With Type 2 Diabetes: A Randomized Noninferiority Trial

**DOI:** 10.1001/jamanetworkopen.2018.0756

**Published:** 2018-07-20

**Authors:** Sharayah Carter, Peter M. Clifton, Jennifer B. Keogh

**Affiliations:** 1 School of Pharmacy and Medical Sciences, University of South Australia, Adelaide, South Australia, Australia; 2 Alliance for Research in Exercise, Nutrition and Activity, University of South Australia, Adelaide, South Australia, Australia

## Abstract

**Importance:**

Intermittent energy restriction is an alternative weight loss method that is becoming popular; however, to date, there are no long-term clinical trials of intermittent energy restriction in patients with type 2 diabetes.

**Objective:**

To compare the effects of intermittent energy restriction (2 days per week) with those of continuous energy restriction on glycemic control and weight loss in patients with type 2 diabetes during a 12-month period.

**Design, Setting, and Participants:**

Adult participants (N = 137) with type 2 diabetes were randomized 1:1 to parallel diet groups (intermittent energy restriction [n = 70] or continuous energy restriction [n = 67]) between April 7, 2015, and September 7, 2017, at the University of South Australia. Medications likely to cause hypoglycemia were reduced at baseline according to the medication management protocol.

**Interventions:**

An intermittent energy restriction diet (500-600 kcal/d) followed for 2 nonconsecutive days per week (participants followed their usual diet for the other 5 days) or a continuous energy restriction diet (1200-1500 kcal/d) followed for 7 days per week for 12 months.

**Main Outcomes and Measures:**

The primary outcome was change in hemoglobin A_1c_ (HbA_1c_) level, with equivalence prespecified by a 90% CI margin of ±0.5%. The secondary outcome was weight loss with equivalence set at ±2.5 kg (±1.75 kg for fat mass loss and ±0.75 kg for fat-free mass loss). All other outcomes were tested for superiority.

**Results:**

Of the 137 randomized participants (77 women and 60 men; mean [SD] age, 61.0 [9.1] years; mean [SD] body mass index, 36.0 [5.8] [calculated as weight in kilograms divided by height in meters squared]; and mean [SD] HbA_1c_ level, 7.3% [1.3%]), 97 completed the trial. Intention-to-treat analysis showed similar reductions in mean (SEM) HbA_1c_ level between the continuous and intermittent energy restriction groups (–0.5% [0.2%] vs –0.3% [0.1%]; *P* = .65), with a between-group difference of 0.2% (90% CI, –0.2% to 0.5%) meeting the criteria for equivalence. Mean (SEM) weight change was similar between the continuous and intermittent energy restriction groups (–5.0 [0.8] kg vs –6.8 [0.8] kg; *P* = .25), but the between-group difference did not meet the criteria for equivalence (–1.8 kg; 90% CI, –3.7 to 0.07 kg), nor did the between-group difference in fat mass (–1.3 kg; 90% CI, –2.8 to 0.2 kg) or fat-free mass (–0.5 kg; 90% CI, –1.4 to 0.4 kg). There were no significant differences between groups in final step count, fasting glucose levels, lipid levels, or total medication effect score at 12 months. Effects did not differ using completers analysis. Hypoglycemic or hyperglycemic events in the first 2 weeks of treatment were similar between the continuous and intermittent energy restriction groups (mean number [SEM] of events, 3.2 [0.7] vs 4.9 [1.4]; *P* = .28), affecting 35% of participants (16 of 46) using sulfonylureas and/or insulin.

**Conclusions and Relevance:**

Intermittent energy restriction is an effective alternative diet strategy for the reduction of HbA_1c_ and is comparable with continuous energy restriction in patients with type 2 diabetes.

**Trial Registration:**

anzctr.org.au Identifier: ACTRN12615000383561

## Key Points

**Question:**

Is intermittent energy restriction comparable to continuous energy restriction for glycemic control in patients with type 2 diabetes?

**Findings:**

In this randomized noninferiority trial, the mean change in hemoglobin A_1c_ level after 12 months in the intermittent energy restriction group (–0.3%) was comparable to that of the continuous energy restriction group (–0.5%), reaching the criterion for equivalence in intention-to-treat and completers analysis.

**Meaning:**

Intermittent energy restriction is comparable to continuous energy restriction for glycemic control in patients with type 2 diabetes.

## Teaser

This randomized noninferiority trial compares the effects of intermittent (2 days per week) energy restricted diet with those of continuous energy restricted diet on glycemic control and weight loss in adults with type 2 diabetes during a 12-month period.

## Introduction

The health care costs of overweight, obesity, and type 2 diabetes are increasing,^1^ and conventional weight-loss diets with daily energy restriction are difficult to adhere to in the long term.^2^ Intermittent energy restriction, short periods of severe energy restriction followed by longer periods of habitual eating, has been suggested as a potential strategy because it offers a reduced burden of dietary restriction and may therefore be more acceptable.^3^ In animal studies, intermittent energy restriction appears to be equally effective as, if not more effective than, continuous energy restriction for the reduction of disease risk factors.^4^ Studies in the healthy overweight and obese population have determined that intermittent energy restriction is an effective method for achieving weight loss comparable to that achieved by continuous energy restriction, with weight loss of 3 to 5 kg after approximately 10 weeks.^5^ Other methods of intermittent energy restriction, such as alternate-day modified fasting, have also demonstrated similar weight loss compared with that achieved by continuous energy restriction.^6^ For individuals with type 2 diabetes, investigators have evaluated the effects of intermittent very-low-calorie diets used within a continuous energy restriction diet compared with continuous energy restriction alone,^7,8,9^ but they have not compared intermittent dieting with continuous dieting. We previously published a pilot trial that demonstrated that a 2-day intermittent energy restriction diet provides an effective alternative method for both glycemic control and weight loss compared with a continuous energy restriction diet over a 3-month period.^10^ The objective of this randomized noninferiority trial was to ascertain the long-term effects of a 2-day intermittent energy restriction diet compared with continuous energy restriction during a 12-month period for patients with type 2 diabetes. We hypothesized that equal improvements to hemoglobin A_1c_ (HbA_1c_) level and weight would occur, as seen in our 3-month pilot trial, thus offering a successful alternative treatment strategy for use in clinical practice.

## Methods

### Study Design

This was a parallel randomized clinical trial, conducted at the Sansom Institute of Health Research, University of South Australia, from April 7, 2015, to September 7, 2017. Reporting in this article is aligned with Consolidated Standards of Reporting Trials (CONSORT) guideline standards, and the full procedure can be found in Supplement 1. Ethics approval was obtained from the University of South Australia Human Research Ethics Committee. Procedures were in accordance with ethical standards, including obtaining written informed consent.

### Participants and Randomization

Participants were recruited using flyers posted in public places and via advertisements in print and broadcast media. Inclusion criteria were adults (≥18 years of age) with type 2 diabetes who were overweight or obese (body mass index ≥27 [calculated as weight in kilograms divided by height in meters squared]) and not pregnant or breastfeeding. Participants reported being otherwise healthy with blood pressure of less than 160/100 mm Hg and no previous weight loss surgery. There was no difference in inclusion criteria between this trial and the pilot trial.^10^ Of the 195 individuals screened for eligibility, 137 were randomized 1:1 to treatment groups, stratified by sex and body mass index (as obese or nonobese), at the initial clinic visit. Randomization was completed using an online-generated random number allocation sequence and was not blinded; participants were allocated to groups by the study dietitian according to the randomization schedule. Participants received an A$25 (US $19) voucher at 3 and 12 months to thank them for their participation; participants were not aware that they would receive a voucher.

### Interventions

Participants randomized to the intermittent energy restriction group followed a diet of 500 to 600 kcal/d for 2 days of the week and followed their usual diet for the other 5 days. The intermittent diet days were mostly nonconsecutive, with a minimum of 50 g of protein per day, in accordance with the very-low-calorie diet guidelines.^11^ See eTable 1 in Supplement 2 for an example meal plan. The continuous energy restriction group followed a diet of 1200 to 1500 kcal/d (30% protein, 45% carbohydrate, and 25% fat).^12^ The total weekly energy estimate was based on the mean adult intake of approximately 2100 kcal/d.^13^ A 75% energy restriction 2 days per week with 5 habitual eating days per week is approximately 11 500 kcal/wk and a 30% daily energy restriction is approximately 10 300 kcal/wk; therefore, both groups had similar dietary energy restrictions recommended. Both groups received written dietary information booklets with portion advice and sample menus; no food or meal replacements were provided. Participants were given digital kitchen scales and encouraged to weigh foods to ensure accuracy of intake. Appointments were with a dietitian (S.C.) and occurred every 2 weeks for the first 3 months and every 2 to 3 months for the final 9 months. Blood glucose control, weight, and diet checklists were reviewed at each visit to assess dietary compliance. The interventions assessed in this trial were no different from the interventions in the pilot trial.^10^

### Medication Management

The medication management protocol was developed after reviewing the literature^14^ and consulting with an endocrinologist (P.M.C.). Management occurred in conjunction with the participant, the study dietitian, and the endocrinologist, as well as with the participant’s medical practitioner. The protocol for a change in medication varied slightly throughout the trial. The initial medication protocol can be found in eTable 2 in Supplement 2 and was modified, primarily owing to hypoglycemia, after the 38th participant commenced the trial. The new protocol required the discontinuation of sulfonylureas and insulin for all participants if the baseline HbA_1c_ level was less than 7% (to convert to proportion of total hemoglobin, multiply by 0.01). If the HbA_1c_ level was higher than 7% but less than 10%, then sulfonylureas and insulin were discontinued on intermittent energy restriction days only, and long-acting insulin was discontinued the night before an intermittent energy restriction day. Medications could be reduced in the continuous energy restriction diet group depending on dose, at the endocrinologist’s discretion. If the HbA_1c_ level was higher than 10%, sulfonylurea medications remained unchanged, but long-acting insulin was decreased by approximately 10 units on intermittent energy restriction diet days only. Although most participants followed the protocol, the endocrinologist worked with each participant individually to ensure the best care. In some cases, participants chose not to follow the protocol or preferred to work with their own medical practitioner for medication adjustment. See eTables 3 and 4 in Supplement 2 for detailed information.

Participants were asked to test and record their fasting blood glucose levels daily before breakfast, with 2 extra readings requested on intermittent energy restriction diet days, including a reading before bedtime. If the blood glucose level was less than 72 mg/dL (to convert to millimoles per liter, multiply by 0.0555), participants were asked to contact the study investigators. Medication changes were then made over the telephone or via email in consultation with the endocrinologist. The records of blood glucose levels were reviewed at each clinic visit, and if the levels were higher than 180 mg/dL, dietary compliance was checked and medication changes were made if necessary. Medication dosages were recorded daily, and the medication effect score (MES) was used to quantify changes. The MES is calculated as (actual drug dose/maximum drug dose) × drug mean adjustment factor.^15^ A higher MES corresponded to a higher dose of diabetes medication, and a reduction in MES corresponded to a reduction in diabetes medication. For example, a 0.5-MES change in the dose of metformin hydrochloride equals a reduction by 1000 mg.

### Outcome Measures

All outcome measures were taken at baseline, 3 months, and 12 months. The primary outcome was change in HbA_1c_ level measured using a DCA (Diabetes Care Analyzer) Vantage Analyzer (Siemens Healthcare Diagnostics) calibrated every 2 weeks. Disposable lancets were used to obtain the sample. The secondary outcome was change in body weight measured (barefoot while wearing light clothing) at each clinic visit on calibrated digital scales in the fasted state (minimum, 8 hours). Body composition was measured by dual-energy x-ray absorptiometry (Lunar iDXA, Getz Healthcare) by a licensed radiation technician. Exploratory outcomes included daily step count, which was monitored via a waistband pedometer (G-sensor, Pocket Pedometer, Walking With Attitude). Participants were encouraged to increase their step count by 2000 from baseline in line with the recommendation of small changes to achieve weight loss.^16^ Participants attended each clinic visit at similar times after at least 1 habitual eating day to match the baseline. Blood samples were obtained to measure fasting glucose and lipid levels, frozen at –80°C, and analyzed at the end of the intervention using Konelab analysis (Konelab 20XTi, Thermo Electron Corporation) at the University of South Australia.

### Statistical Analysis

Sample size was calculated using the standard deviation of the change in HbA_1c_ level from our pilot data.^10^ We required a minimum sample size of 104 participants to demonstrate equivalence between groups: *P* < .05 with 80% power and a 90% CI boundary of ±0.5%. For weight, a very similar number was required, using a boundary limit of ±2.5 kg (±1.75 kg for fat mass and ±0.75 kg for fat-free mass). The predefined margin of equivalence for HbA_1c_ level, weight, fat mass, and fat-free mass was based on clinical relevance. A 0.5% change in HbA_1c_ level is considered to be a significant clinical change and is half the expected change achieved by most hypoglycemic drugs.^17^ All other measures were exploratory, and we had no hypothesis with regard to equivalence, so only superiority tests were performed.

Analyses were performed using SPSS, version 21 (IBM SPSS Statistical Software), and data are shown as mean (standard error of the mean) values unless otherwise stated. A 2-tailed *P* < .05 was considered statistically significant. Independent samples *t* tests and Pearson χ^2^ tests were used to analyze differences between groups at baseline. Change over time, differences between treatments, and time by treatment interactions were assessed on an intention-to-treat basis, including data from all 137 participants who underwent randomization under a missing-at-random assumption tested using a linear mixed model and from 97 completers using repeated-measures analysis of variance. Factors significant in a Pearson correlation were entered into stepwise linear regression to determine independent factors associated with major outcome measures at 12 months. Graphs were generated using Microsoft Excel 2010 for Windows (Microsoft Inc), and data were reported as the mean between-group difference in change, with a 2-sided 90% confidence interval, including the equivalence margin. The full statistical analysis plan is available in Supplement 1.

## Results

**Figure 1.**
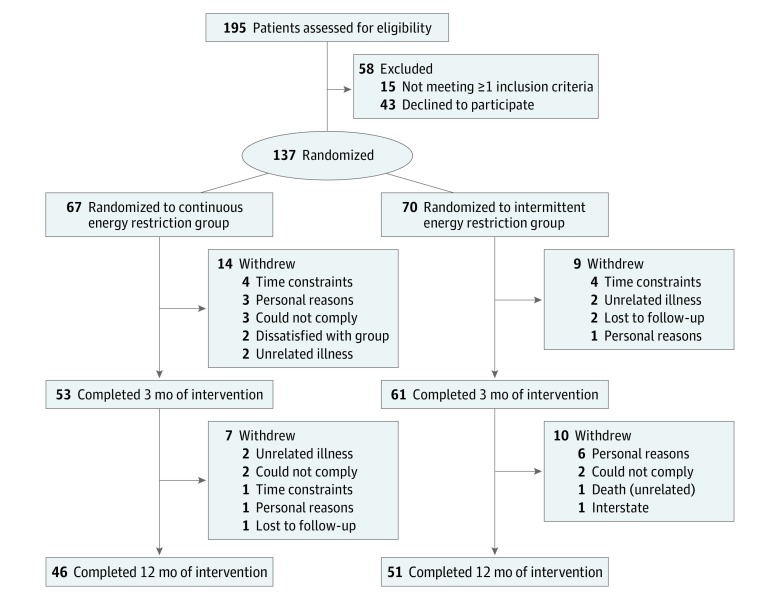
Flow Diagram

Of the 195 participants screened, 137 Australian adults with type 2 diabetes (77 women and 60 men; mean [SD] age, 61.0 [9.1] years; mean [SD] body mass index, 36.0 [5.8]; and mean [SD] HbA_1c_ level, 7.3% [1.3%]) were randomly assigned to diet groups. Of these 137 participants, 67 were randomized to the continuous energy restriction group and 70 were randomized to the intermittent energy restriction group. Ninety-seven participants (70.8%) completed the study (Figure 1), and the dropout rates were similar in both groups (21 participants [31.3%] in the continuous energy restriction group and 19 participants [27.1%] in the intermittent energy restriction group; *P* = .71). Baseline characteristics were comparable between groups (Table 1).

**Table 1.** Baseline Characteristics of Participants^a^

Characteristic	Mean (SD) Value				
	Continuous Energy Restriction Group (n = 67)	Intermittent Energy Restriction Group (n = 70)	All Participants (N = 137)	Participants Who Completed Study (n = 97)	Participants Who Did Not Complete Study (n = 40)
Age, y	61.0 (9.2)	61.0 (9.0)	61.0 (9.1)	62.0 (8.8)	59.0 (9.6)
Sex, No. (%)					
Female	38 (56.7)	39 (55.7)	77 (56.2)	48 (49.5)	29 (72.5)
Male	29 (43.3)	31 (44.3)	60 (43.8)	49 (50.5)	11 (27.5)
Duration of diabetes, y	8.1 (6.5)	7.9 (5.9)	8.0 (6.2)	7.9 (6.2)	8.3 (6.2)
Glycemic control					
HbA_1c_, %	7.5 (1.4)	7.2 (1.2)	7.3 (1.3)	7.1 (1.2)	7.8 (1.4)
FPG, mg/dL	158 (44)	149 (39)	153 (42)	154 (42)	148 (42)
Diabetes medications, No. (%)					
Diet	20 (29.9)	18 (25.7)	38 (27.7)	27 (27.8)	11 (27.5)
OHA	39 (58.2)	43 (61.4)	82 (59.9)	57 (58.8)	25 (62.5)
Metformin	43 (64.2)	46 (65.7)	89 (65.0)	65 (67.0)	24 (60.0)
DPP-4 inhibitors	11 (16.4)	9 (12.9)	20 (14.6)	13 (13.4)	7 (17.5)
SGLT2 inhibitors	4 (6.0)	4 (5.7)	8 (5.8)	3 (3.1)	5 (12.5)
GLP-1 agonists	4 (6.0)	1 (1.4)	5 (3.6)	4 (4.1)	1 (2.5)
Sulfonylureas	12 (17.9)	18 (25.7)	30 (21.9)	21 (21.6)	9 (22.5)
Insulin	14 (20.9)	14 (20.0)	28 (20.4)	21 (21.6)	7 (17.5)
Medication effect score^b^					
OHA	1.4 (0.8)	1.3 (0.8)	1.4 (0.8)	1.3 (0.8)	1.4 (0.7)
Insulin	1.5 (1.1)	1.8 (1.1)	1.6 (1.1)	1.5 (0.9)	2.1 (1.6)
Total	1.8 (1.1)	1.7 (1.3)	1.8 (1.2)	1.7 (1.2)	1.8 (1.1)
CVD risk markers					
Lipid-lowering, No. (%)	41 (61.2)	46 (65.7)	87 (63.5)	64 (66.0)	23 (57.5)
Total cholesterol, mg/dL	195 (64)	179 (48)	186 (56)	187 (60)	181 (23)
HDL cholesterol, mg/dL	46 (17)	47 (14)	47 (16)	46 (16)	50 (13)
LDL cholesterol, mg/dL	116 (50)	107 (43)	111 (46)	111 (49)	108 (24)
Triglycerides, mg/dL	168 (127)	129 (62)	147 (99)	151 (104)	116 (40)
Body weight and composition					
Weight, kg	102 (17)	100 (19)	101 (18)	100 (17)	103 (19)
BMI	37 (5.7)	35 (5.8)	36 (5.8)	35 (5.6)	37 (6.1)
Total body fat, %	44 (6.6)	42 (7.3)	43 (7.0)	42 (7.1)	45 (6.2)
Total fat mass, kg	42 (9.1)	40 (9.4)	41 (9.3)	40 (9.2)	43 (9.4)
Total fat-free mass, kg	54 (9.5)	54 (9.8)	54 (9.6)	55 (9.6)	52 (9.5)
Android fat, %^c^	52 (5.6)	51 (6.1)	52 (5.9)	51 (6.1)	53 (5.1)
Android fat mass, kg	4.8 (1.2)	4.4 (1.1)	4.6 (1.2)	4.5 (1.1)	4.8 (1.3)
Android fat-free mass, kg	4.3 (0.9)	4.2 (0.8)	4.2 (0.8)	4.2 (0.8)	4.3 (1.0)
VAT, kg	2.5 (0.9)	2.2 (0.9)	2.3 (0.9)	2.4 (0.9)	2.4 (0.9)
Physical activity					
Activity count, steps/d	5889 (2893)	6800 (3187)	6363 (3071)	6327 (3088)	7180 (2206)

Abbreviations: BMI, body mass index (calculated as weight in kilograms divided by height in meters squared); CVD, cardiovascular disease; DPP-4, dipeptidyl peptidase 4; FPG, fasting plasma glucose; GLP-1, glucagon-like peptide 1 receptor; HbA_1c_, hemoglobin A_1c_; HDL, high-density lipoprotein; LDL, low-density lipoprotein; OHA, oral hypoglycemic agents; SGLT2, sodium-glucose cotransporter 2; VAT, visceral adipose tissue.

SI conversion factors: To convert HbA_1c_ to proportion of total hemoglobin, multiply by 0.01; FPG to millimoles per liter, multiply by 0.0555; total, HDL, and LDL cholesterol to millimoles per liter, multiply by 0.0259; and triglycerides to millimoles per liter, multiply by 0.0113.

^a^ Data were analyzed using independent samples *t* test (for continuous variables) and χ^2^ test (for categorical variables) and given as mean (SD) value.

^b^ Medication effect score: (actual drug dose/maximum drug dose) × drug mean adjustment factor.

^c^ Percentage fat of tissue in the android region.

**Figure 2.**
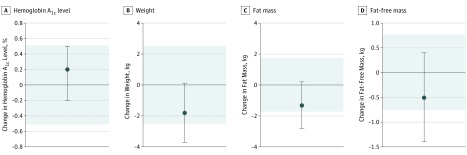
Mean Between-Group Difference in Change in Hemoglobin A_1c_ Level, Weight, and Body Composition for the Intermittent vs Continuous Groups (Intention-to-Treat Analysis) To convert hemoglobin A_1c_ to proportion of total hemoglobin, multiply by 0.01. Error bars indicate 2-sided 90% confidence intervals. Tinted area indicates zone of equivalence.

From baseline to 12 months, the mean (SEM) HbA_1c_ level was reduced significantly, with no difference between treatment groups (–0.5% [0.2%] in the continuous energy restriction group vs –0.3% [0.1%] in the intermittent energy restriction group; *P* = .65), and the results did not differ using completers analysis (eTable 5 in Supplement 2). For the primary outcome, the mean between-group difference in the change in HbA_1c_ level was 0.2% (90% CI, –0.2% to 0.5%) and was contained within the equivalence margin of ±0.5%, confirming equivalence (Figure 2). There was an increase in the mean (SEM) HbA_1c_ level in both groups between 3 and 12 months (0.3% [0.1%]), with an overall decrease of 0.4% (0.1%) (*P* < .001) at 12 months. In stepwise linear regression, the baseline HbA_1c_ level accounted for 35% and the change in visceral adipose tissue accounted for 17% of the variance in the change in HbA_1c_ level at 12 months (adjusted *r*^2^ = 0.5; *P* < .001), with no effect of treatment group. Participants with a baseline HbA_1c_ level higher than 8% had the greatest mean (SEM) change (–1.4% [0.2%]; *P* < .001), and participants with a baseline HbA_1c_ level of less than 6% had almost no change (–0.03% [0.05%]; *P* = .50) at 12 months.

The total mean (SEM) MES decreased significantly by time (–0.5 [0.1]; *P* < .001), was similar in both groups (–0.3 [0.1] in the continuous energy restriction group vs –0.6 [0.1] in the intermittent energy restriction group; *P* = .11), and was correlated with weight change at 12 months (*r* = 0.3; *P* = .009). The mean (SEM) MES for oral hypoglycemic agents decreased significantly by time (–0.3 [0.1]; *P* < .001) and was similar in both groups (–0.2 [0.1] in the continuous energy restriction group vs –0.3 [0.1] in the intermittent energy restriction group; *P* = .45), but, for insulin, the decrease was significantly greater in the intermittent energy restriction group owing to the extra insulin reduction required on intermittent diet days (–0.3 [0.1] in the continuous energy restriction group vs –1.2 [0.2] in the intermittent energy restriction group; *P* = .006). Most of the changes to medications were made before 3 months, with small changes between 3 and 12 months (total mean [SEM] MES, –0.1 [0.05]; *P* = .02), with no difference between groups (with a total mean [SEM] MES of –0.06 [0.05] in the continuous energy restriction group vs –0.2 [0.08] in the intermittent energy restriction group; *P* = .28). Thirty-five percent of participants (16 of 46) using sulfonylureas and/or insulin experienced glycemic events (the mean [SEM] number of events was 3.2 [0.7] in the continuous energy restriction group and 4.9 [1.4] in the intermittent energy restriction group; *P* = .28) in the first 2 weeks of treatment. Eight of 46 participants (17%) (2 in the continuous energy restriction group and 6 in the intermittent energy restriction group) experienced hypoglycemia (mean [SEM] number of events, 2.4 [0.6]) with no differences between groups (the mean [SEM] number of events was 2.0 [1.0] in the continuous energy restriction group vs 2.5 [0.8] in the intermittent energy restriction group; *P* = .74). All participants who experienced hypoglycemic events either reported events before starting treatment or were unsure. One participant in the intermittent energy restriction group who experienced hypoglycemia during the first 2 weeks of treatment did not lose weight during this time. Hyperglycemia occurred in 10 of 46 participants (22%; 3 in the continuous energy restriction group and 7 in the intermittent energy restriction group) in the first 2 weeks (mean [SEM] number of events, 5.1 [1.4]), with no difference between groups (the mean [SEM] number of events was 4.0 [0.6] in the continuous energy restriction group vs 5.6 [2.0] in the intermittent energy restriction group; *P* = .47). Three participants did not lose weight during this period (1 in the continuous energy restriction group and 2 in the intermittent energy restriction group). Four participants chose not to follow the protocol or chose to follow their own medical practitioner’s advice (eTables 3 and 4 in Supplement 2).

The analysis of the secondary outcome showed that the mean [SEM] weight reduction was significant over time but not by treatment (–5.0 [0.8] kg in the continuous energy restriction group vs –6.8 [0.8] kg in the intermittent energy restriction group; *P* = .25), and the results did not differ using completer analysis (eTable 5 in Supplement). The mean between-group difference in weight change was –1.8 kg (90% CI, –3.7 to 0.07 kg), which is outside the prespecified boundary of ±2.5 kg, so statistical equivalence has not been shown (Figure 2). Twenty percent of participants (19 of 97) lost 5% to 10% of their body weight, and 22% (21 of 97) lost more than 10% of their body weight, with no difference between groups. Both groups lost significant weight between baseline and 3 months, which was maintained at 12 months (mean [SEM] weight reduction from 3 to 12 months was 0.4 [0.5] kg in the continuous energy restriction group and –0.2 [0.6] kg in the intermittent energy restriction group; *P* = .48). Weight change at 2 weeks was associated with weight loss at 12 months (*r* = 0.6; *P* < .001). A small subgroup of participants (21 in both groups) continued to lose weight during the 12 months of the study (mean [SEM] weight reduction was –8.4 [1.2] kg in the continuous energy restriction group and –12.5 [1.8] kg in the intermittent energy restriction group; *P* = .07), and these participants maintained the lower HbA_1c_ level seen at 3 to 12 months (mean [SEM] level, 0.6% [0.2%]; *P* < .001) with no difference by treatment. Participants who attended all scheduled visits lost significantly more weight than did participants who did not (mean [SEM], –7.6 [0.9] kg vs –4.0 [1.1] kg; *P* = .01), with a trend toward a greater change in HbA_1c_ level (mean [SEM] level, –0.5% [0.1%] vs –0.2% [0.1%]; *P* = .09). In stepwise linear regression, change in step count at 3 months accounted for 9%, and the baseline step count accounted for 5% of the change in weight at 12 months (adjusted *r*^2^ = 0.1; *P* < .001), with no effect of treatment group. All measures of body composition decreased significantly over time, with no difference between groups (Table 2). The mean between-group differences in change were –1.3 kg (90% CI, –2.8 to 0.2 kg) for fat mass and –0.5 kg (90% CI, –1.4 to 0.4 kg) for fat-free mass, which are outside the prespecified boundaries of ±1.75 kg for fat mass and ±0.75 kg for fat-free mass, so statistical equivalence has not been shown (Figure 2). The total loss of fat-free mass was significantly greater in the intermittent energy restriction group using completers analysis (eTable 5 in Supplement 2) but did not differ between groups when expressed as a mean (SEM) percentage of weight lost (31% [8.5%] in the continuous energy restriction group and 37% [9.6%] in the intermittent energy restriction group; *P* = .61).

**Table 2.** Primary, Secondary, and Exploratory Outcomes From Baseline to 12 Months for Intermittent vs Continuous Groups (Intention-to-Treat Analysis)^a^

Variable	Mean (SEM) [95% CI]	*P* Value for Time	Mean (SEM) [95% CI]		*P* Value for Diet by Time
			Continuous Restriction Group	Intermittent Restriction Group	
Primary outcome					
HbA_1c_, %	−0.4 (0.1) [−0.6 to −0.2]	<.001	−0.5 (0.2) [−0.8 to −0.2]	−0.3 (0.1) [−0.6 to −0.08]	.65
Secondary outcomes					
Weight, kg	−5.9 (0.6) [−7.1 to −4.8]	<.001	−5.0 (0.8) [−6.6 to −3.5]	−6.8 (0.8) [−8.5 to −5.1]	.25
BMI	−2.1 (0.2) [−2.5 to −1.7]	<.001	−1.9 (0.3) [−2.4 to −1.3]	−2.3 (0.3) [−2.9 to −1.7]	.43
Total body fat, %^b^	−1.8 (0.4) [−2.5 to −1.1]	<.001	−1.6 (0.3) [−2.2 to −0.9]	−2.3 (0.6) [−3.5 to −1.1]	.20
Total fat mass, kg^b^	−4.1 (0.5) [−5.0 to −3.2]	<.001	−3.4 (0.6) [−4.6 to −2.2]	−4.7 (0.7) [−6.1 to −3.4]	.20
Total fat-free mass, kg^b^	−1.8 (0.3) [−2.4 to −1.3]	<.001	−1.6 (0.4) [−2.3 to −0.8]	−2.1 (0.4) [−2.9 to −1.4]	.11
Android fat, %^b^^,^^c^	−3.8 (1.1) [−5.9 to −1.7]	<.001	−2.0 (1.2) [−4.4 to −0.5]	−5.6 (1.7) [−9.0 to −2.1]	.23
Android fat mass, kg^b^	−0.8 (0.2) [−1.2 to −0.5]	<.001	−0.6 (0.2) [−1.1 to −0.2]	−1.1 (0.2) [−1.5 to −0.6]	.37
Android fat-free mass, kg^b^	−0.3 (0.1) [−0.5 to −0.04]	.05	−0.3 (0.2) [−0.6 to −0.02]	−0.3 (0.2) [−0.6 to −0.08]	.75
VAT, kg^b^	−0.2 (0.06) [−0.3 to −0.1]	<.001	−0.2 (0.09) [−0.4 to −0.02]	−0.2 (0.09) [−0.4 to −0.08]	.42

Abbreviations: BMI, body mass index (calculated as weight in kilograms divided by height in meters squared); HbA_1c_, hemoglobin A_1c_; VAT, visceral adipose tissue.

SI conversion factor: To convert HbA_1c_ to proportion of total hemoglobin, multiply by 0.01.

^a^ Data were included for 137 participants (67 in the continuous energy restriction group and 70 in the intermittent energy restriction group) unless otherwise stated: mean (SEM) and 95% CI were estimated using an intention-to-treat analysis with a linear mixed model.

^b^ For a total of 128 participants (64 in the continuous energy restriction group and 64 in the intermittent energy restriction group) with weight greater than 130 kg or who declined dual-energy x-ray absorptiometry scan.

^c^ Percentage fat of tissue in the android region.

The exploratory outcomes of mean (SEM) fasting glucose (–18.1 [4.6] mg/dL) and serum lipid levels (total cholesterol level, –13.0 [3.9] mg/dL [to convert to millimoles per liter, multiply by 0.0259]; low-density lipoprotein cholesterol level, –11.1 [3.3] mg/dL [to convert to millimoles per liter, multiply by 0.0259]; triglycerides level, –16.0 [8.5] mg/dL [to convert to millimoles per liter, multiply by 0.0113]; and high-density lipoprotein cholesterol level, –1.4 [1.5] mg/dL [to convert to millimoles per liter, multiply by 0.0259]) improved with weight loss (*P* < .001), with no differences between groups. The increase in mean (SEM) steps per day was significantly greater in the continuous energy restriction group than the intermittent energy restriction group at 12 months (1183 [516] steps vs 524 [483] steps; *P* = .02), but the total step count was still similar in both groups (7071 [529] steps in the continuous energy restriction group vs 7312 [496] steps in the intermittent energy restriction group; *P* = .52). Effects did not differ using completers analysis.

## Discussion

To our knowledge, this is the first randomized clinical trial of this duration comparing intermittent energy restriction with continuous energy restriction in patients with type 2 diabetes. The results demonstrated that a 2-day intermittent energy restriction diet is comparable to a continuous energy restriction diet for improvements in glycemic control, confirming our pilot data.^10^ Equivalence was demonstrated for change in HbA_1c_ level; however, given the large variability in weight reduction, equivalence could not be demonstrated for weight loss or changes in body composition. Intermittent energy restriction may be superior to continuous energy restriction for weight reduction, although a sample population of more than 300 participants would be required to demonstrate superiority with 80% power.

The major factor associated with change in HbA_1c_ level was baseline HbA_1c_ level, and because participants had relatively well-controlled type 2 diabetes, our capacity to see a large change was limited. It has been well demonstrated that the magnitude of the effect of an intervention on glycemic control depends on baseline HbA_1c_ level.^18^ The change in HbA_1c_ level in our trial is comparable to the change seen in trials with similar baseline HbA_1c_ level, such as the Look AHEAD (Action for Health in Diabetes) study.^19^ Long duration of diabetes was associated with a higher baseline HbA_1c_ level, which resulted in a greater reduction in HbA_1c_ level. Relative weight maintenance from 3 to 12 months probably contributed to the loss of effect on HbA_1c_ level during this period because participants who continued to lose weight maintained improved HbA_1c_ levels. The slight but nonsignificant difference in change in HbA_1c_ level between groups may be because the 5 days of nonrestricted habitual eating for those in the intermittent energy restriction group had no specific emphasis on improved dietary choices as were provided to those in the continuous energy restriction group.

Intermittent energy restriction is safe for people who have either diet-controlled type 2 diabetes or are using medication that is not likely to cause hypoglycemia. For people using sulfonylureas and/or insulin, intermittent energy restriction requires medication changes and regular monitoring, especially in the initial stages. Patients need to be able to contact their medical practitioner for further medication changes if they experience a hypoglycemic event. In our trial, hypoglycemic events occurred only in participants who reported experiencing hypoglycemia before starting treatment or who were uncertain.

In this pragmatic trial, every effort was made to try and replicate the real-world environment; therefore, no meal replacements or foods were provided. Considering this fact, acceptability of the energy restriction plans was very high, with only 2 participants from the intermittent energy restriction group withdrawing owing to headaches brought on by hunger. Overall compliance to both diets was excellent during the first 3 months of treatment (90% in the continuous energy restriction group and 97% in the intermittent energy restriction group; *P* = .21), after which the compliance rates decreased in both groups (49% in the continuous energy restriction group and 44% in the intermittent energy restriction group; *P* = .62). Anecdotally, participants in the intermittent energy restriction group reported that although they were not following the protocol consistently after 3 months, they found that they could use it effectively to prevent weight gain because the energy restriction involved only 2 days. Intermittent dieting has been shown to improve the efficiency of weight loss compared with continuous dieting,^20^ although we did not find this result in the present study. The continuous energy restriction group found weight loss maintenance more difficult because, if they were not following the diet on a daily basis, they would regain weight owing to increased energy intake. Weight loss was greater in a subgroup of participants who attended all scheduled clinic visits with the study dietitian compared with participants who missed sessions. The benefits of behavioral support,^21^ specifically support provided by a dietitian,^22^ as well as the effects of lifestyle interventions,^23^ are noted in the literature. There was a significant difference in loss of total fat-free mass, which was greater in the intermittent energy restriction group for completers. This difference did not occur in our pilot trial, nor is it seen in the literature on intermittent energy restriction in overweight and obese individuals.^5,10^ We do not believe that this finding is clinically relevant, considering the greater weight loss in the intermittent energy restriction group at 12 months (approximately 2 kg) and because the total fat-free mass percentage change did not differ between groups.

### Limitations

This study has some limitations. First, as indicated, this population had well-controlled type 2 diabetes, which limits generalizability. Second, we acknowledge that medication adjustments can cause problems with interpreting changes in HbA_1c_ level. Changes to medications were similar between groups, except for reductions in insulin, which were greater in the intermittent energy restriction group and may have limited changes in HbA_1c_ level. Third, participants had more contact time with the dietitian than is usual in the clinical setting, especially in the first 3 months, which may have affected the results. Finally, only finger-prick testing was used to monitor blood glucose levels, and it is likely that hyperglycemic and hypoglycemic events went undetected.^24^ Future research should use continuous blood glucose monitoring systems for more accurate results.

## Conclusions

Intermittent energy restriction is an effective alternative diet strategy for the reduction of HbA_1c_ level comparable to continuous energy restriction in patients with type 2 diabetes, and it may be superior to continuous energy restriction for weight reduction. Intermittent energy restriction is acceptable for most patients with type 2 diabetes, and safety can be ensured for patients who are not using glycemic agents likely to cause hypoglycemia. For patients using sulfonylureas and/or insulin, regular monitoring is paramount. The relative maintenance of results between the 3- and 12-month periods may reflect the importance of support for behavioral change, which was provided more frequently in the first 3 months of treatment and resulted in greater change if participants attended all appointments.

## Notes

Supplement 1. **Trial Protocol**

Supplement 2. **eTable 1.** Intermittent Energy Restriction Example Meal Plan **eTable 2.** Initial Medication Protocol **eTable 3.** Medication Changes Using First Protocol **eTable 4.** Medication Changes Using Second Protocol **eTable 5.** Primary, Secondary, and Exploratory Outcomes From Baseline to 12 Months for Intermittent vs Continuous Groups, Completers Analysis

## References

[1] Colagiuri S, Lee CM, Colagiuri R, The cost of overweight and obesity in Australia. Med J Aust. 2010;192(5):-. 20201759 10.5694/j.1326-5377.2010.tb03503.x

[2] Middleton KR, Anton SD, Perri MG Long-term adherence to health behavior change. Am J Lifestyle Med. 2013;7(6):395-404. 27547170 10.1177/1559827613488867 PMC4988401

[3] Johnstone A Fasting for weight loss: an effective strategy or latest dieting trend? Int J Obes (Lond). 2015;39(5):727-733. 25540982 10.1038/ijo.2014.214

[4] Longo VD, Mattson MP Fasting: molecular mechanisms and clinical applications. Cell Metab. 2014;19(2):181-192. 24440038 10.1016/j.cmet.2013.12.008 PMC3946160

[5] Seimon RV, Roekenes JA, Zibellini J, Do intermittent diets provide physiological benefits over continuous diets for weight loss? a systematic review of clinical trials. Mol Cell Endocrinol. 2015;418(pt 2):153-172. 26384657 10.1016/j.mce.2015.09.014

[6] Trepanowski JF, Kroeger CM, Barnosky A, Effect of alternate-day fasting on weight loss, weight maintenance, and cardioprotection among metabolically healthy obese adults: a randomized clinical trial. JAMA Intern Med. 2017;177(7):930-938. 28459931 10.1001/jamainternmed.2017.0936 PMC5680777

[7] Williams KV, Mullen ML, Kelley DE, Wing RR The effect of short periods of caloric restriction on weight loss and glycemic control in type 2 diabetes. Diabetes Care. 1998;21(1):2-8. 9538962 10.2337/diacare.21.1.2

[8] Wing RR, Blair E, Marcus M, Epstein LH, Harvey J Year-long weight loss treatment for obese patients with type II diabetes: does including an intermittent very-low-calorie diet improve outcome? Am J Med. 1994;97(4):354-362. 7942937 10.1016/0002-9343(94)90302-6

[9] Wing RR, Marcus MD, Salata R, Epstein LH, Miaskiewicz S, Blair EH Effects of a very-low-calorie diet on long-term glycemic control in obese type 2 diabetic subjects. Arch Intern Med. 1991;151(7):1334-1340. 2064484

[10] Carter S, Clifton PM, Keogh JB The effects of intermittent compared to continuous energy restriction on glycaemic control in type 2 diabetes; a pragmatic pilot trial. Diabetes Res Clin Pract. 2016;122:106-112. 27833048 10.1016/j.diabres.2016.10.010

[11] Baker S, Jerums G, Proietto J Effects and clinical potential of very-low-calorie diets (VLCDs) in type 2 diabetes. Diabetes Res Clin Pract. 2009;85(3):235-242. 19560834 10.1016/j.diabres.2009.06.002

[12] CSIRO. CSIRO total wellbeing diet online. https://www.csiro.au/en/Research/Health/CSIRO-diets/CSIRO-Total-Wellbeing-Diet-Online. Accessed August 1, 2014.

[13] Australian Bureau of Statistics *National Nutrition Survey: Selected Highlights, Australia, 1995*. Canberra, Australia: Australian Bureau of Statistics; 1998.

[14] Carter S, Clifton PM, Keogh JB Intermittent energy restriction in type 2 diabetes: a short discussion of medication management. World J Diabetes. 2016;7(20):627-630. 28031781 10.4239/wjd.v7.i20.627 PMC5155237

[15] Mayer SB, Jeffreys AS, Olsen MK, McDuffie JR, Feinglos MN, Yancy WS Jr Two diets with different haemoglobin A1c and antiglycaemic medication effects despite similar weight loss in type 2 diabetes. Diabetes Obes Metab. 2014;16(1):90-93. 23911112 10.1111/dom.12191 PMC3867584

[16] Hill JO, Wyatt HR, Reed GW, Peters JC Obesity and the environment: where do we go from here? Science. 2003;299(5608):853-855. 12574618 10.1126/science.1079857

[17] Sherifali D, Nerenberg K, Pullenayegum E, Cheng JE, Gerstein HC The effect of oral antidiabetic agents on A1C levels: a systematic review and meta-analysis. Diabetes Care. 2010;33(8):1859-1864. 20484130 10.2337/dc09-1727 PMC2909079

[18] Pillay J, Armstrong MJ, Butalia S, Behavioral programs for type 2 diabetes mellitus: a systematic review and network meta-analysis. Ann Intern Med. 2015;163(11):848-860. 26414227 10.7326/M15-1400

[19] Pi-Sunyer X, Blackburn G, Brancati FL, ; Look AHEAD Research Group Reduction in weight and cardiovascular disease risk factors in individuals with type 2 diabetes: one-year results of the Look AHEAD trial. Diabetes Care. 2007;30(6):1374-1383. 17363746 10.2337/dc07-0048 PMC2665929

[20] Byrne NM, Sainsbury A, King NA, Hills AP, Wood RE Intermittent energy restriction improves weight loss efficiency in obese men: the MATADOR study. Int J Obes (Lond). 2018;42(2):129-138. 28925405 10.1038/ijo.2017.206 PMC5803575

[21] Cradock KA, ÓLaighin G, Finucane FM, Diet behavior change techniques in type 2 diabetes: a systematic review and meta-analysis. Diabetes Care. 2017;40(12):1800-1810. 29162585 10.2337/dc17-0462

[22] Møller G, Andersen HK, Snorgaard O A systematic review and meta-analysis of nutrition therapy compared with dietary advice in patients with type 2 diabetes. Am J Clin Nutr. 2017;106(6):1394-1400. 29092883 10.3945/ajcn.116.139626

[23] Johansen MY, MacDonald CS, Hansen KB, Effect of an intensive lifestyle intervention on glycemic control in patients with type 2 diabetes: a randomized clinical trial. JAMA. 2017;318(7):637-646. 28810024 10.1001/jama.2017.10169 PMC5817591

[24] Weber KK, Lohmann T, Busch K, Donati-Hirsch I, Riel R High frequency of unrecognized hypoglycaemias in patients with type 2 diabetes is discovered by continuous glucose monitoring. Exp Clin Endocrinol Diabetes. 2007;115(8):491-494. 17853331 10.1055/s-2007-984452

