# Supplementary material for: Effect of Intermittent Compared With Continuous Energy Restricted Diet on Glycemic Control in Patients With Type 2 Diabetes: A Randomized Noninferiority Trial
Source: JAMA Netw Open. 2018 Jul 20;1(3):e180756. doi: 10.1001/jamanetworkopen.2018.0756 (PMC6324303; doi:10.1001/jamanetworkopen.2018.0756)
Supplement: Supplement 1. — Trial Protocol [file jamanetwopen-1-e180756-s001.pdf]

Statistical Analysis Plan for 5:2 Diabetes Study: The effects of intermittent compared to continuous energy restriction on glycaemic control in type 2 diabetes: A randomised control trial.

Registered: Australian New Zealand Clinical Trials Registry. **Number:** ACTRN12615000383561 **Date:** 27/04/2015.

Version: 3.0 **Date:** April 2015

Protocol Version	Updated SAP Version	Section No. Changed	Description of and reason for change	Date Changed
1.0	2.0	Medication Management	New Medication Management Protocol	Aug 2015
1.0	3.0	Sample size	Pilot trial SD data available	December 2015

Researchers: Jennifer Keogh, PhD (Primary Supervisor), Peter Clifton, MD, PhD (Secondary Supervisor), Sharayah Carter BND, PhD Candidate.

Responsibilities: SC, PMC and JBK designed research and will analysed data. SC will conduct the research and write the paper. PMC and JBK (guarantor) will have primary responsibility for final content and critically review the manuscript.

Name: Jennifer Keogh

Signature:

Name: Peter Clifton

Signature

Table of Contents

1 Background 1

2 Aims and Objectives 1

2.1 Secondary aims: 1

2.2 Hypothesis 2

3 Methods 2

3.1 Trial Design 2

3.2 interim Analysis 2

3.3 Ethics Approval 2

3.4 Randomisation 2

3.5 Sample Size 2

3.6 Final Analysis 3

3.7 Primary Outcome Measure 3

3.8 Secondary Outcome Measures 3

3.9 Exploratory Outcome Measures 3

3.10 Statistical Analysis 4

3.11 Adherence and Protocol 4

3.12 Recruitment 4

3.13 Inclusion and Exclusion Criteria 5

3.14 Medication Management 5

3.14.1 Medication Effect Score 6

1 Background

The prevalence of overweight and obesity is increasing. In 1995, 56.3% of the Australian adult population were overweight or obese and in 2012 this statistic had increased to 62.8%[1]. Overweight and obesity are significant health issues as they are major risk factors in the development of chronic preventable diseases such as (type 2 diabetes mellitus) T2DM. Approximately 90% of people with T2DM are overweight or obese[2] therefore, eliminating obesity from the population could potentially reduce the incidence of T2DM by over 40%[3]. Weight loss improves metabolic outcomes, with a reduction of only 5-10% body weight leading to improvements in glycaemic control, lipid profile and blood pressure[4]. Currently the most common form of weight loss is continuous energy restriction (CER), a moderate daily energy reduction of approximately 25% of energy requirements. Recently however, intermittent energy restriction (IER) has gained popularity. Intermittent energy restriction is an alternative weight loss method involving partial dieting; limited days of severe energy restriction (~75%) followed by habitual eating and may prove useful for individuals who find CER difficult to maintain. Recent studies have shown IER to be comparable to CER in achieving weight loss in overweight and obese populations[5-8] with the most common weight loss being 3-5kg after approximately 10 weeks[9]. However, no trials have been published comparing IER with CER in people with T2DM. Only intermittent use of low or very low energy diets used within a CER diet compared to CER diet alone[10-13]. Therefore, research is needed to demonstrate the effects of IER on glycaemic control and weight loss in type 2 diabetes.

2 Aims and Objectives

The overall aim of this research is to determine the effects of a 2-day IER diet on glycaemic control (HbA1c) over 12 months in T2DM. The protocol will initially be piloted over 3 months to establish efficacy of treatment and a safe medication management protocol.

2.1 SECONDARY AIMS:

- To investigate change in weight and body composition, as well as exercise, appetite and lipid profile after 12 months of treatment.
- To establish effective medication management protocol to allow for the safe use of IER as a treatment method.

2.2 HYPOTHESIS

The null hypothesis is that there will be no difference in glycaemic improvements at 12 months between the two treatment groups. Furthermore changes in weight, body composition, exercise, appetite and lipid profile will also be similar at 12 months.

3 Methods

3.1 TRIAL DESIGN

The trial is a parallel-randomised clinical trial. Treatment allocation is a 1:1 ratio. Patients are randomised to either continuous or intermittent energy restriction.

3.2 INTERIM ANALYSIS

One formal statistical interim analysis is planned on all outcome measures 3 months into recruitment to determine efficacy, safety and a formal sample size calculation for a long-term trial (12 months). Pilot participants will have reached the first time point of assessment (3 months).

3.3 ETHICS APPROVAL

Ethics approval was obtained from the University of South Australia Human Research Ethics Committee (Application No: 0000033918). Reporting of adverse events (related/non-related) to ethics is a requirement and will be completed by SC or JBK. E.g. hypoglycaemia as a result of treatment.

3.4 RANDOMISATION

Participants will be randomised 1:1 to treatment groups, stratified by gender and BMI (obese or non-obese). Randomisation will be completed using an online generated random number allocation sequence (www.randomization.com) and will not be blinded.

3.5 SAMPLE SIZE

Recruit 100 participants based on previous studies[7].

After the pilot trial a sample size was calculated using the standard deviation of the change in HbA1c[14]. We required a minimum sample size of 104 participants to demonstrate equivalence between diet groups; $P < .05$ with 80% power and a 90% CI boundary of ±0.5%. For weight a very similar number will be required using a boundary limit of ±2.5kg (±1.75kg

for fat mass and ±0.75kg for lean mass). The margin of equivalence was decided based on a clinical relevance. We will endeavour to recruit 30% above sample size calculation to account for dropouts.

3.6 FINAL ANALYSIS

Final analysis will occur after the last participant has completed to 12 months (estimated date: October 2017). Publication of results will occur in 2018.

3.7 PRIMARY OUTCOME MEASURE

- All outcome measures will be measured fasting (min. 8hrs), after at least one habitual eating day for IER group to match the baseline. Important time-points for all outcome measures include, baseline, 3 and 12 months and will be reported as change by time and time by treatment.
- HbA1c (%) will be measured using a DCA Vantage Analyzer (Siemens). A disposable lancet will be used to complete the finger-prick after the area was sanitized with a disposable alcohol wipe. The machine will be calibrated fortnightly.

3.8 SECONDARY OUTCOME MEASURES

- Weight (kg) measured on calibrated digital scales (no shoes, light clothing) at fortnightly fasting visits until 3 months, then every 3 months until 12 months.
- Lean body mass/fat mass measured using Duel-Energy X-ray Absorptiometry.

3.9 EXPLORATORY OUTCOME MEASURES

- Fasting blood samples will be taken by venepuncture to measure fasting plasma glucose (mmol/L), lipid levels (mmol/L) and gamma-glutamyl transferase. 300 microliter aliquots of plasma and serum with be taken and stored at -80°C until analysis. Plasma glucose and serum total cholesterol, HDL cholesterol, triglycerides, will be measured using a Konelab 20XTi automatic analyser (Thermo Electron Corporation, Louisville, CO, USA) with reagents from Thermo Fisher Scientific (Melbourne, Australia). LDL cholesterol will be calculated using the Friedewald formula (total cholesterol - HDL cholesterol) – (triglycerides x 0.45)[15].
- Participants will be given a waistband pedometer (G-sensor Accelerometer Pedometer) at baseline and will be asked to monitor and record their steps daily, making no changes to their current exercise levels. Average steps will be calculated at the second clinic visit and all participants will be asked to increase their step count

by 2000 ('small changes') and maintain this increase over the length of the trial [16]. All participants will be encouraged to meet their individual goal.

- Hunger scores, which will be monitored via a validated visual analogue scales (VAS) and will be used to assess participants' appetite markers i.e. hunger, fullness, satisfaction, appetite, using a validated Likert scale survey [17].

3.10 STATISTICAL ANALYSIS

Analyses will be performed using SPSS V21. A 2-tailed P<.05 will be considered statistically significant.

Continuous data will be summarised by mean, SD or SEM. Independent samples t-tests and Chi-squared will be used to analyse differences between groups at baseline. Patients will be described with respect to age, gender, HbA1c, year since diagnosis, weight, height, fasting glucose, lipid profile, medication use and medication change (MES), steps, appetite.

Change over time, differences between treatments and time by treatment interactions will be assessed using repeated measures ANOVA for completers (attended final outcome visit [12 months]). Intention-to-treat analysis will be performed on all participants randomised to treatment groups using linear mixed modelling under a missing-at-random assumption. Pearson correlations will be used to analyse correlations and correlated variables will be entered into stepwise linear regression to determine independent predictors. Graphs will be generated using Microsoft Excel and will include mean, SD or SEM and CI for change data.

3.11 ADHERENCE AND PROTOCOL

Attending outcome appointments, specifically the final assessment visit at 12 months will be considered adherence to protocol and will be described in the published manuscript as 'completers'. Further analysis of participants who continued to lose weight will also occur to assess compliance to protocol and will be reported. Participants will only be asked to withdraw if they choose to follow a different diet method.

3.12 RECRUITMENT

Participants will be recruited from the general population via flyers posted in public places or by advertisement in local newspapers or other general media, e.g. online, radio etc. Recruitment agency may be used. A CONSORT flow diagram will be used to summarise people screened, eligible, randomised, receiving their allocated treatment, withdrawing/lost to follow-up.

3.13 INCLUSION AND EXCLUSION CRITERIA

**Inclusion Criteria**	Adults >18 years<br>BMI >27kg/m^2<br>T2DM (Diet Control, OHA or Insulin)<br>Not pregnant or breast feeding
**Exclusion Criteria**	No history of weight loss surgery or weight loss >5kg in past 3 months<br>Blood Pressure (>160/100mmHg)<br>Women who are pregnant or breast feeding or wish to become pregnant<br>Serious illness not otherwise managed (e.g. cancer, liver or renal disease)<br>Drinking > 2 standard alcoholic drinks per day and not able/willing to decrease

3.14 MEDICATION MANAGEMENT

Professor Peter Clifton, endocrinologist, will manage medication changes as follows:

All participants will be asked to test and record their fasting blood glucose levels daily, i.e. before breakfast, with the addition of 2 extra readings requested on intermittent diet days, which must include a before bed reading.

- <4mmol/L participants will be asked to contact study investigators via phone or email for medication changes.
- >10mmol/L dietary compliance will be checked at clinic visit and if necessary medication changes were made.

Medication Protocol

HbA1c	OHA (Sulphonylureas)	Insulin
**<8%**	Discontinue at baseline for both	IER: Halve insulin dose on intermittent days<br>CER: Reduce by ~10 units/day

	groups	** Added during pilot trial: If insulin doses before bed were considered to be too high in preparation for an intermittent diet day, before bed doses were also decreased.*
**>8%**	N/A	**IER:** Reduce by ~10 units on IER days only

Adjusted Medication Protocol

**HbA1c**	**OHA (Sulphonylureas)**	**Insulin**
**<7%**	Discontinue at baseline for both groups	Discontinue at baseline for both groups
**>7-10%**	Discontinue on IER days only	**IER:** Discontinued on IER days only and long acting insulin discontinued the night before IER day. Insulin will not be resumed until a full day's caloric intake is planned (if taken in the morning) or achieved (if taken in the evening). **CER:** ~10 unit reduction depending on dose
**>10**	Continues	**IER:** ~10 unit reduction on IER days only

3.14.1 Medication Effect Score

Medication effect score (MES) assesses overall utilization of OHA and insulin. MES is calculated as MES = (actual drug dose/maximum drug dose)*drug mean adjustment factor[18]. In this equation adjustment factors equate to the expected decrease in HbA1c achieved by the drug as a monotherapy; a summary is provided in the table below[19]. MES will be calculated at each time point to provide medication change over time data.

Medication cost savings will be calculated using prices listed on the Australian Pharmaceutical Benefits Scheme.

Medication maximum dose and adjustment factors

Drugs	Maximum Dose	Adjustment Factor
Metformin	3000mg	1.5
DPP4	100mg/5mg	0.65
SGLT2 (e.g. Forxiga)	10mg	0.8
Sulphonylurea	120mg	1.5
Exenatide (e.g. Byetta)	20ug	2.5
Insulin	1unit/kg	2.5

DPP4, dipeptidyl peptidase-4 inhibitor; SGLT2, sodium/glucose cotransporter 2

References

1. Australian Bureau of Statistics. *Australian Health Survey: First Results 2011-12; Overweight and Obesity. 2012.*
2. Dixon JB, Browne JL, Mosely KG, et al. Severe obesity and diabetes self-care attitudes, behaviours and burden: implications for weight management from a matched case-controlled study. Results from Diabetes MILES—Australia. *Diabetic Medicine.* 2014;31(2):232-240.
3. Baker S, Jerums G, Proietto J. Effects and clinical potential of very-low-calorie diets (VLCDs) in type 2 diabetes. *Diabetes research and clinical practice.* 2009;85(3):235-242.
4. Maggio CA, Pi-Sunyer FX. Obesity and type 2 diabetes. *Endocrinology and Metabolism Clinics of North America.* 2003;32(4):805-822.
5. Harvie M, Wright C, Pegington M, et al. The effect of intermittent energy and carbohydrate restriction v. daily energy restriction on weight loss and metabolic disease risk markers in overweight women. *The British journal of nutrition.* 2013;110(8):1534-1547.
6. Harvie MN, Pegington M, Mattson MP, et al. The effects of intermittent or continuous energy restriction on weight loss and metabolic disease risk markers: a randomized trial in young overweight women. *International journal of obesity (2005).* 2011;35(5):714-727.
7. Keogh JB, Pedersen E, Petersen KS, Clifton PM. Effects of intermittent compared to continuous energy restriction on short-term weight loss and long-term weight loss maintenance. *Clinical Obesity.* 2014;4(3):150-156.
8. Varady KA. Intermittent versus daily calorie restriction: which diet regimen is more effective for weight loss? *Obesity reviews : an official journal of the International Association for the Study of Obesity.* 2011;12(7):e593-601.
9. Seimon RV, Roekenes JA, Zibellini J, et al. Do intermittent diets provide physiological benefits over continuous diets for weight loss? A systematic review of clinical trials. *Molecular and cellular endocrinology.* 2015.
10. Redmon JB, Raatz SK, Reck KP, et al. One-year outcome of a combination of weight loss therapies for subjects with type 2 diabetes a randomized trial. *Diabetes Care.* 2003;26(9):2505-2511.
11. Williams KV, Mullen ML, Kelley DE, Wing RR. The effect of short periods of caloric restriction on weight loss and glycemic control in type 2 diabetes. *Diabetes care.* 1998;21(1):2-8.
12. Wing RR, Blair EH, Marcus MD, Epstein LH, Harvey J. Year-Long Weight Loss Treatment for Obese Patients With Type II Diabetes: Does Including an Intermittent Very-Low-Calorie Diet Improve Outcome? *The Journal of American Medicine.* 1994;97:354-362.
13. Wing RR, Marcus MD, Salata R, Epstein LH, Miaskiewicz S, Blair EH. Effects of a very-low-calorie diet on long-term glycemic control in obese type 2 diabetic subjects. *Arch Intern Med.* 1991;151(7):1334-1340.
14. Carter S, Clifton PM, Keogh JB. The effects of intermittent compared to continuous energy restriction on glycaemic control in type 2 diabetes; a pragmatic pilot trial. *Diabetes research and clinical practice.* 2016;122:106-112.

15. Friedewald WT, Levy RI, Fredrickson DS. Estimation of the concentration of low-density lipoprotein cholesterol in plasma, without use of the preparative ultracentrifuge. *Clinical chemistry.* 1972;18(6):499-502.

16. Hill JO, Wyatt HR, Reed GW, Peters JC. Obesity and the environment: where do we go from here? *Science (New York, NY).* 2003;299(5608):853-855.

17. Flint A, Raben A, Blundell JE, Astrup A. Reproducibility, power and validity of visual analogue scales in assessment of appetite sensations in single test meal studies. *International journal of obesity and related metabolic disorders : journal of the International Association for the Study of Obesity.* 2000;24(1):38-48.

18. Mayer SB, Jeffreys AS, Olsen MK, McDuffie JR, Feinglos MN, Yancy WS, Jr. Two diets with different haemoglobin A1c and antiglycaemic medication effects despite similar weight loss in type 2 diabetes. *Diabetes, obesity & metabolism.* 2014;16(1):90-93.

19. Nathan DM, Buse JB, Davidson MB, et al. Medical Management of Hyperglycemia in Type 2 Diabetes: A Consensus Algorithm for the Initiation and Adjustment of Therapy: A consensus statement of the American Diabetes Association and the European Association for the Study of Diabetes. *Diabetes Care.* 2009;32(1):193-203.
