# Supplementary material for: Effect of Intermittent Compared With Continuous Energy Restricted Diet on Glycemic Control in Patients With Type 2 Diabetes: A Randomized Noninferiority Trial
Source: JAMA Netw Open. 2018 Jul 20;1(3):e180756. doi: 10.1001/jamanetworkopen.2018.0756 (PMC6324303; doi:10.1001/jamanetworkopen.2018.0756)
Supplement: Supplement 2. — eTable 1. Intermittent Energy Restriction Example Meal Plan eTable 2. Initial Medication Protocol eTable 3. Medication Changes Using First Protocol eTable 4. Medication Changes Using Second Protocol eTable 5. Primary, Secondary, and Exploratory Outcomes From Baseline to 12 Months for Intermittent vs Continuous Groups, Completers Analysis [file jamanetwopen-1-e180756-s002.pdf]

Supplementary Online Content

Carter S, Clifton PM, Keogh JB. Effect of intermittent compared with continuous energy restricted diet on glycemic control in patients with type 2 diabetes: a randomized noninferiority trial. *JAMA Netw Open*. 2018;1(3):e180756.
doi:10.1001/jamanetworkopen.2018.0756

eTable 1. Intermittent Energy Restriction Example Meal Plan
eTable 2. Initial Medication Protocol
eTable 3. Medication Changes Using First Protocol
eTable 4. Medication Changes Using Second Protocol
eTable 5. Primary, Secondary, and Exploratory Outcomes From Baseline to 12 Months for Intermittent vs Continuous Groups, Completers Analysis

This supplementary material has been provided by the authors to give readers additional information about their work.

© 2018 Carter S et al. *JAMA Network Open.*

eTable 1. Intermittent Energy Restriction Example Meal Plan

	**500kcals**	**600kcals**
**Breakfast**	1 serve of fruit (150g)<br>+<br>1 tub of diet yoghurt	
**Lunch**	Small tin of tuna in spring water<br>+<br>1 cup of salad	Add: 1 serve of breads/cereals e.g. 'thin style' wrap
**Dinner**	100g of cooked chicken breast<br>(1 teaspoon of oil for cooking)<br>+<br>1.5 cups cooked low carbohydrate vegetables	

Note: 2 litres of fluid was recommended. Diet products were allowed e.g. diet jelly.

© 2018 Carter S et al. *JAMA Network Open.*

eTable 2. Initial Medication Protocol

HbA1c	Sulphonylureas	Insulin
**<8%**	Discontinue at baseline for both groups	**CER:** Reduce by ~10 units/day<br>**IER:** Halve insulin dose on intermittent days<br>**If insulin dose before bed was considered to be too high in preparation for an intermittent diet day, before bed dose was also decreased.*
**>8%**	N/A	**IER:** Reduce by ~10 units on IER days only

Abbreviations: CER, continuous energy restriction; IER, intermittent energy restriction; ~, approximately.

© 2018 Carter S et al. *JAMA Network Open.*

eTable 3. Medication Changes Using First Protocol[a]

Grp.	Med. Category	HbA_{1c} (%)	Initial Med. Dose	Initial Change	Protocol	Wt loss after first 2wks	Event during first 2wks	Change after first 2wks	Events (3wks-3mths)	Changes before 3mths	Event (3mth-12mths)	Changes after 3mths	Final Med.
CER	OHA	6.2	150mg	Discont.	Yes	Yes	n/a	n/a	n/a	n/a	n/a	n/a	n/a
IER	OHA	6.7	60mg	Discont.	Yes	Yes	n/a	n/a	n/a	n/a	Hypers (non-IER d)	No OHA on IER d	No OHA on IER d
IER	OHA	7.3	60mg	Discont.	Yes	No	n/a	n/a	n/a	Restarted OHA (pt MP)	Hypo (IER d)	Discont. OHA on IER d	No OHA on IER d
IER	OHA	7.5	120mg	Discont.	Yes	Yes	n/a	n/a	Hypers (both d)	Added back half dose (60mg)	Hypo (both d)	Discont. OHA	No OHA
CER	Insulin (LONG + RAPID) + OHA	7.6	LONG 25u, RAPID 12u, OHA 60mg	LONG 12u, RAPID 6u, Discont. OHA	Yes	Yes	Hypers (x3)	LONG 16u, RAPID 7u (pt MP)	n/a	n/a	Hypers	LONG 22u, RAPID 11u (pt MP), LONG 27u, RAPID 8u (pt MP)	LONG 27u, RAPID 8u
CER	Insulin (MIX)	8.6	25/45u	None	Yes	Yes	Hypo (x3)	20/30u	Hypo	20/25u, 20/20u	Hypo	15/25u, 15/30u (levels inc.)	15/30u
CER	Insulin (MIX)	9	88/58u	None	Yes	No	n/a	n/a	W/D				
CER	Insulin	9.7	30/22u	None	Yes	Yes	Hypo (x1)	25/17u	n/a	20/15u (low), 17/10u (low), 20/10u (levels inc.)	n/a	20/5u (low)	20/5u

© 2018 Carter S et al. *JAMA Network Open.*

Grp.	Med. Category	HbA1c (%)	Initial Med. Dose	Initial Change	Protocol	Wt loss after first 2wks	Event during first 2wks	Change after first 2wks	Events (3wks-3mths)	Changes before 3mths	Event (3mth-12mths)	Changes after 3mths	Final Med.
IER	Insulin (LONG + RAPID)	7.1	LONG 40u, RAPID 15/15u	LONG 20u, RAPID 7/7u on IER d	Yes	Yes	Hypo (x2) (both d)	LONG 20u on IER d and LONG 30u, RAPID 7/7u on non-IER d	n/a	LONG 30u only on non-IER d (low)	Hypo (both d)	LONG 15u on IER d and 20u on non-IER d, LONG 10u on IER d and 20u non-IER d (low)	LONG 10u on IER d, 20u non-IER d
IER	Insulin (MIX)	7.3	80/75u	40/38u on IER d	Yes	No	Hypo (x3) (IER d)	20/20u on IER d	Hypo (IER d)	20/10u on IER d	W/D		
IER	Insulin	8.8	6/41u	6/20u night before and on IER d	No, > required	Yes	Hypo (x1) (IER d)	0/10u on IER d	n/a	n/a	W/D		
IER	Insulin	8.8	10/50u	10/30u on IER d and 25u night before IER d	No, > required	Yes	n/a	n/a	Hypo (IER d)	10/30u non-IER d, 10/10u night before and IER d	n/a	n/a	10/30u non-IER d, 10/10u night before and IER d
IER	Insulin (LONG + RAPID)	11.5	LONG 28/15/28u, RAPID 28/15/28u	None	Yes	W/D							

[a]Recommendations made by the study's endocrinologist unless otherwise specified. First protocol, eTable 2.

Abbreviations: Grp, group; Med, medication; HbA_{1c}, glycated hemoglobin; Wt, weight; wks, weeks; mths, months; CER, continuous energy restriction; IER, intermittent energy restriction; OHA, oral hypoglycemic agent (gliclazide unless otherwise stated); Discont, discontinue; hypers, hyperglycemia; hypo, hypoglycemia; low, 72-106mg/dL; inc. increasing; W/D, withdrew; d, day/s; pt, participant; MP, medical practitioner; Insulin, long-acting insulin (insulin glargine); LONG; long-acting insulin (insulin glargine); MIX, NovaMix; RAPID, NovaRapid; u, units

© 2018 Carter S et al. *JAMA Network Open.*

eTable 4. Medication Changes Using Second Protocol[a]

Grp.	Med. Category	HbA_{1c} (%)	Initial Med. Dose	Initial Change	Protocol	Wt loss after first 2wks	Event during first 2wks	Change after first 2wks	Events (3wks-3mths)	Changes before 3mths	Event (3mth-12mths)	Changes after 3mths	Final Med.
CER	OHA	6.1	OHA 120mg	Discont. OHA, pt MP decrease to 60mg	No. At pt MP advice, < required	Yes	n/a	n/a	n/a	n/a	n/a	n/a	60mg OHA
CER	OHA	7.4	OHA 20mg	None	Yes	Yes	n/a	n/a	n/a	n/a	W/D		
CER	OHA	7.7	OHA 80mg	None	Yes	No	n/a	n/a	n/a	n/a	W/D		
CER	OHA	8.3	OHA 60mg	None	Yes	No	n/a	n/a	n/a	n/a	n/a	n/a	n/a
CER	OHA	8.4	OHA 60mg	None	Yes	Yes	n/a	n/a	n/a	n/a	W/D		
CER	OHA	9	OHA 120mg	None	Yes	Yes	n/a	n/a	n/a	60mg OHA (low)	W/D		
CER	OHA	10.9	OHA 80mg	None	Yes	W/D							
IER	OHA (GP)	6	OHA 1mg	Discont. OHA	Yes	Yes	n/a	n/a	n/a	n/a	n/a	n/a	n/a
IER	OHA	6.2	OHA 60mg	Discont. OHA	Yes	Yes	Hypers (x2) (non-IER d)	n/a	n/a	n/a	n/a	n/a	n/a
IER	OHA	7	OHA 60mg	No OHA on IER d	Yes	No	n/a	n/a	Hypos (non-IER d)	30mg OHA on non-IER d	W/D		
IER	OHA (GP)	7	OHA 2mg	No OHA on IER d	Yes	Yes	n/a	n/a	n/a	Discont. OHA (low)	n/a	n/a	No OHA

© 2018 Carter S et al. *JAMA Network Open.*

Grp.	Med. Category	HbA_{1c} (%)	Initial Med. Dose	Initial Change	Protocol	Wt loss after first 2wks	Event during first 2wks	Change after first 2wks	Events (3wks-3mths)	Changes before 3mths	Event (3mth-12mths)	Changes after 3mths	Final Med.
IER	OHA	7.3	OHA 60mg	No OHA on IER d	Yes	No	Hypers (x2) (both d)	Restart OHA	W/D				
IER	OHA	7.6	OHA 120mg	No OHA on IER d	Yes	Yes	Hypos (x2) (non-IER d) and Hypers (x5) (non-IER d)	Decrease to 60mg OHA on non-IER d	n/a	n/a	n/a	n/a	60mg OHA on non-IER d
IER	OHA	7.8	OHA 120mg	No OHA on IER d	Yes	No	n/a	n/a	W/D				
IER	OHA	7.9	OHA 120mg	No OHA on IER d	Yes	Yes	n/a	n/a	n/a	n/a	Hypers (both d)	Restart OHA	OHA 120mg
IER	OHA	9.2	OHA 60mg	No OHA on IER d	Yes	Yes	n/a	n/a	n/a	Discont. OHA completely (low)	n/a	n/a	No OHA
IER	OHA	9.5	OHA 60mg	No OHA on IER d	Yes	Yes	n/a	n/a	n/a	n/a	n/a	n/a	No OHA on IER d
CER	Insulin (LONG + RAPID)	6.2	LONG 36u, RAPID 12/12u	LONG 30u, RAPID discont.	No, < required	Yes	n/a	n/a	n/a	LONG 28u (low)	n/a	LONG 22u (low)	22u
CER	Insulin	6.9	20u	None	No, < required	Yes	n/a	n/a	n/a	n/a	n/a	n/a	n/a
CER	Insulin	7.1	12u	10u	No, all pt would agree too, < required	No	n/a	n/a	n/a	9u (low)	n/a	8u (low)	

© 2018 Carter S et al. *JAMA Network Open.*

Grp.	Med. Category	HbA_{1c} (%)	Initial Med. Dose	Initial Change	Protocol	Wt loss after first 2wks	Event during first 2wks	Change after first 2wks	Events (3wks-3mths)	Changes before 3mths	Event (3mth-12mths)	Changes after 3mths	Final Med.
CER	Insulin (MIX) + OHA	7.5	22/30u, OHA 120mg	15/20u, Discont. OHA	No, > required	Yes	n/a	n/a	Hyper	15/25u	Hypers	17/27u	17/27u
CER	Insulin + OHA (GP)	7.8	26u, OHA 4mg	None	No, < required	Yes	n/a	n/a	Hypo	20u, 18u, 16u	n/a	14u, 12u, 10u (low)	10u
CER	Insulin	8.3	36u	None	Yes	W/D							
CER	Insulin	8.5	80/34u	None	Yes	Yes	Hypers (x5)	n/a (pt MP advice)	Hypers	n/a (pt MP advice)	Hypers	n/a (pt MP advice)	n/a (pt MP advice)
CER	Insulin	8.8	30u	None	Yes	No	Hypers (x4)	n/a	n/a	n/a	Hypers	35u, 38u, 40u	40u
CER	Insulin	9.3	75/75u	None	Yes	Yes	n/a	n/a	n/a	n/a	n/a	70/70u (low)	70/70u
CER	Insulin + OHA (GP)	10	25u, OHA 4mg	None	Yes	Yes	n/a	n/a	Low	20u	W/D		
IER	Insulin (MIX) + OHA	5.7	45/45u, OHA 60mg	10/10u, no OHA on IER d, 25/25u on non-IER d	No, < required	Yes	n/a	n/a	Hyper	25/30 non-IER d	Hypers	10/15u on IER d, 15/30u on non-IER d	10/15u on IER d, 15/30u non-IER d
IER	Insulin + OHA	7.5	16u, OHA 60mg	No insulin the night before or on IER d, no OHA on IER d	Yes	Yes	Hypers (x4) (non-IER d)	18u non-IER d	n/a	n/a	n/a	16u non-IER d (low)	16u non-IER d

© 2018 Carter S et al. *JAMA Network Open.*

Grp.	Med. Category	HbA_{1c} (%)	Initial Med. Dose	Initial Change	Protocol	Wt loss after first 2wks	Event during first 2wks	Change after first 2wks	Events (3wks-3mths)	Changes before 3mths	Event (3mth-12mths)	Changes after 3mths	Final Med.
IER	Insulin + OHA	7.6	70/70u, OHA 60mg	10/10u, no OHA on IER d and 10u the night before on IER d	No, all pt would agree too, < required	Yes	Hypos (x6) (both d), Hypers (x2) (non-IER d)	50/50u non-IER d	Hypos (both d)	40/40u non-IER d and no insulin night before IER d or on IER d	Hypos (non-IER d)	30/30u on non-IER d and no insulin night before IER d or on IER d, 20/20u	20/20u non-IER d
IER	Insulin	7.7	40u	30u on IER d	No. Pts MP, < required	Yes	n/a	n/a	n/a	n/a	n/a	n/a	30u
IER	Insulin (MIX)	7.8	40/40u	No insulin on IER d	Yes	No	n/a	n/a	n/a	n/a	Hypers (non-IER d)	65/65u on non-IER d, 30/30u on non-IER d (low)	30/30u
IER	Insulin (MIX + RAPID)	7.9	44/24u MIX, 6u RAPID as req.	No MIX night before or on IER d	Yes	No	Hypers (x14) (both d)	10u of RAPID on IER d as req.	n/a	n/a	n/a	n/a	n/a
IER	Insulin	8.7	32u	No insulin night before or on IER d	Yes	Yes	Hypo (x1) (non-IER d)	25u on non-IER d	Hypo (non-IER d)	20u on non-IER d	W/D		
IER	Insulin + OHA (GP)	9	26u, OHA 4mg	5u night before IER d and none on IER d	No, < required	Yes	Hypers (x12) (both d)	10u night before IER d	Hypo (IER d)	Decrease to 5u night before IER d	Hypers (both d)	10u night before IER d, 20u on non-IER d (low)	20u non-IER d, 10u night before and none on IER d
IER	Insulin + OHA (GP)	9.8	34u, OHA 4mg	22u on IER d	No, < required	Yes	n/a	17u on IER d (low)	n/a	15u on IER d (low)	n/a	12u on IER d (low)	12u

[a]Recommendations made by the study's endocrinologist unless otherwise specified. Second protocol detailed in manuscript.

© 2018 Carter S et al. *JAMA Network Open.*

Abbreviations: Grp, group; Med, medication; HbA_{1c}, glycated hemoglobin; Wt, weight; wks, weeks; mths, months; CER, continuous energy restriction; IER, intermittent energy restriction; OHA, oral hypoglycemic agent (gliclazide unless otherwise stated); GP, glimepiride; Discont, discontinue; hypers, hyperglycemia; hypo, hypoglycemia; low, 72-106mg/dL; d, day/s; W/D, withdrew; pt, participant; MP, medical practitioner; Insulin, long-acting insulin (insulin glargine); LONG; long-acting insulin (insulin glargine); MIX, NovaMix; RAPID, NovaRapid; u, units

© 2018 Carter S et al. *JAMA Network Open.*

eTable 5. Primary, Secondary and Exploratory Outcomes From Baseline to 12 Months for Intermittent vs Continuous Groups (Completers Analysis)[a]

Variable	Mean (SEM) [95% CI]				
	**All Participants (n = 97)**	**P Value for Time**	**Continuous**	**Intermittent**	**P Value for Diet by Time**
**Primary Outcome**					
HbA1c, %	-0.4 (0.09) [-0.6 to -0.1]	<.001	-0.4 (0.2) [-0.8 to -0.05]	-0.3 (0.1) [-0.6 to -0.01]	.62
**Secondary Outcomes**					
Weight, kg[b]	-6.1 (0.7) [-7.8 to -4.3]	<.001	-5.0 (0.8) [-7.0 to -3.0]	-7.1 (1.1) [-9.9 to -4.4]	.15
BMI, kg/m^2	-2.1 (0.2) [-2.7 to -1.5]	<.001	-1.8 (0.3) [-2.5 to -1.1]	-2.5 (0.4) [-3.4 to -1.6]	.19
Total Body Fat, %[c]	-2.2 (0.4) [-3.3 to -1.1]	<.001	-1.7 (0.4) [-2.7 to -0.6]	-2.8 (0.8) [-4.7 to -0.9]	.21
Total Fat Mass, kg[c]	-4.4 (0.6) [-5.9 to -3.0]	<.001	-3.7 (0.7) [-5.4 to -1.9]	-5.2 (0.9) [-7.5 to -2.9]	.22
Total FF Mass, kg[c]	-1.8 (0.2) [-2.3 to -1.3]	<.001	-1.3 (0.3) [-2.1 to -0.5]	-2.2 (0.3) [-2.9 to -1.5]	.02
Android Fat, %[c]	-3.3 (0.7) [-5.0 to -1.6]	<.001	-2.1 (0.5) [-3.4 to -0.8]	-4.5 (1.2) [-7.6 to -1.4]	.07
Android Fat Mass, kg[c]	-0.7 (0.1) [-0.9 to -0.5]	<.001	-0.6 (0.1) [-0.9 to -0.3]	-0.9 (0.1) [-1.2 to -0.5]	.14
Android FF Mass, kg[c]	-0.2 (0.04) [-0.3 to -0.1]	<.001	-0.3 (0.05) [-0.2 to 0.1]	-0.2 (0.06) [-0.2 to 0.1]	.80
VAT, kg[c]	-0.2 (0.07) [-0.4 to -0.1]	<.001	-0.2 (0.09) [-0.4 to -0.05]	-0.3 (0.1) [-0.5 to -0.05]	.38

Abbreviations: HbA_{1c}, glycated hemoglobin; BMI, body mass index; FF, fat free; VAT, visceral adipose tissue.

[a]Data were included for 97 participants (CER group: n=46; IER group: n=51) unless otherwise stated: mean (SEM) and [95% CI] were estimated with repeated-measures ANOVA.
[b]Total analysed: n=94 (CER group: n=44; IER group: n=50) 3 participants did not attend final appointment, GP sent HbA1c results.
[c]Total analysed: n=81 (CER group: n=39; IER group: n=42) weight >130kg or declined DEXA scan.

© 2018 Carter S et al. *JAMA Network Open.*
